# Evaluating Consumer Nutrition Environment in Food Deserts and Food Swamps

**DOI:** 10.3390/ijerph18052675

**Published:** 2021-03-07

**Authors:** He Jin, Yongmei Lu

**Affiliations:** 1 School of Geosciences, University of South Florida, 4202 E Fowler Ave, Tampa, FL 33620, USA; 2 Department of Geography, Texas State University, 601 University Dr., San Marcos, TX 78666, USA; YL10@txstate.edu

**Keywords:** convenience stores, food insecurity, grocery stores and supermarkets

## Abstract

This research examines the consumer nutrition environment in the selected neighborhoods identified as food deserts, food swamps, and food oases in Austin, Texas, by considering food availability, food price, food quality, and food labeling. A food auditing instrument M-TxNEA-S (He Jin, San Marcos, TX, USA) was developed to capture the unique dietary culture and food preferences in Texas. A total of 93 food items in 14 grocery stores and supermarkets (GS) and 32 convenience stores (CS) were surveyed. The GS in food swamps and food oases were found to offer significantly more healthy foods than the CS. The availability of healthy food in the GS in the food swamps and food oases is significantly higher than that of the GS from the food deserts; CS in the three neighborhoods did not exhibit a significant difference in healthy food availability. There was no significant difference between the price for the healthy items (lower fat, lower calorie, and whole grain) and that for the regular food options. No significant difference was found for food quality or food labeling between the stores from the different types of neighborhoods. The GS in food deserts are small grocery stores carrying limited ranges of foods. The establishment of larger food stores in the food deserts might not be very rewarding, but opening more small grocery stores with healthier options may alleviate food issues.

## 1. Introduction

In 2011, 37.1% of residents in Austin, Texas were overweight, and 27.0% were obese. By 2016, the percentages had risen by 21%. Studies examined the effects of physical inactivity and diet on overweight and obesity [1,2,3], and these studies found that diet has a greater effect on weight outcomes [4]. Several recent studies explored the impact of the diet nutrition environment on dietary behaviors and weight status [5,6,7,8,9,10].

Among different frameworks for investigating the nutrition environment, Glanz and colleagues’ framework is the most renowned [2]. This framework suggests that the nutrition environment consists of two aspects: community nutrition environment and consumer nutrition environments. The community nutrition environment focuses on the effect of the number and location of food outlets on health outcomes, which has been explored by many scholars and researchers [5,6,7,10,11]. The consumer nutrition environment refers to consumers’ experience in food stores, including food availability, food affordability, food quality, and other in-store characteristics. The consumer nutrition environment has been less studied compared to the community nutrition environment [2].

Food availability measures the presence or absence of food items in stores [12]. Greater availability of healthy foods (e.g., fruits and vegetables) is positively related to the consumption of these items [13]. Food affordability mainly refers to food costs relative to income. Food cost was identified as an essential barrier to healthy eating for low-income communities [14] and households [15]. Food quality refers to characteristics of food that are acceptable to consumers such as appearance, texture, and packaging. Poor food quality (e.g., withered or bruised fresh produce, rotting meat, and expired canned foods) could deter food purchasing behaviors [16], and therein impose an adverse effect on diet quality and health outcomes. Food labeling provides detailed information on food nutrient facts. Appropriate food labeling facilitates consumers to make better decisions. Food labeling positively predicted dietary quality and dietary behaviors [17,18] and also projected a decrease in adults’ long-term body weight [19]. However, in comparison with the other three aspects, food labeling has been less explored in consumer nutrition environment assessment.

Studies of the consumer nutrition environment have often concerned whether people from deprived neighborhoods and minority-dominated communities experience low availability of healthy foods, high food prices, and low food quality across the different types of stores [1,2,20,21]. These studies have been motivated by the deprivation amplification hypothesis [22]; the core concept of this hypothesis is “the disadvantageous environments magnify individual vulnerability, resulting in (built-) environmental characteristics more detrimental to health in deprived areas” [3]. This hypothesis emphasizes the amplification of disadvantaged social environment or context on individuals or household deprivation. Therefore, the consumer nutrition environment was studied by contrasting different types of neighborhoods, high-income vs. low-income or White-dominated vs. African American (or Hispanic)-dominated neighborhoods.

Food deserts were created by the suburbanization of grocery stores and supermarkets. In the 1970s, American cities were in an “urban crisis”; urban areas became impoverished after the middle class moved away from cities [23]. Population changes resulted in a significant loss of grocery stores and supermarkets in urban neighborhoods. People who lived in food deserts were often in low socioeconomic status and could not afford healthy and nutritious foods. These unfavorable factors created severe barriers to healthy eating. There are various definitions of food deserts, and we adopted Stein’s definition [24] since it takes both geographic access to food outlets and sociodemographic deprivation into consideration. In this study, food deserts are defined as individuals or groups having barriers accessing nutritional and affordable foods in socially deprived areas [24]. On the contrary, a food oasis is “a place where self-sustaining and innovative practices are developed to empower inhabitants of food deserts to have better access to healthy eating environments and foods [25]”. This definition seems reasonable, but it misses an important component—socioeconomic status (SES). In food oases, residents not only have abundant access to healthy food outlets such as supermarkets, grocery stores, farmers’ markets, and even community gardens [26,27], but also families typically have a higher SES, resulting in a higher prevalence of affording healthier and fresher options [28]. As a result, we defined food oases as areas where residents had plentiful access to healthful foods in socially privileged areas. Currently, turning food deserts into food oases had become the primary goal for some organizations and health agencies that aim at promoting human well-being [29].

The limited access to healthy foods in food deserts is a useful proxy for under-nutrition. However, the issue in developed countries such as the U.S. is over-consumption of nutrition. The overwhelming unhealthy food in low-income neighborhoods is a more severe problem than in food deserts [24]. Therefore, the term “food swamp” is used to describe the areas where high-calorie and energy-dense foods swamp out healthy options in socially deprived areas [24]. Note that various “food swamp” definitions were developed in the food access literature; we used Stein’s [24] definition due to the same reason as the food deserts. Convenience stores in food swamps provide a selection of unhealthy foods, which usually are cheaper than their healthy options. Many residents in food swamps are of low socioeconomic status and tend to buy energy-dense but less expensive foods, leading to increased caloric intake, lower intake of fruits and vegetables, and elevating the risk of obesity [30,31]. Nevertheless, no studies have examined the consumer nutrition environment in food deserts and food swamps. Additionally, there is a general lack of thorough investigations on the interaction effect of store types and income levels on the consumer nutrition environment [1,2,20].

Through conducting a food store survey in Austin, Texas, this study compared the consumer nutrition environment across the selected food swamp, food desert, and food oasis neighborhoods; both grocery stores and supermarkets (GS) and convenience stores (CS) were investigated. We hypothesize that GS would have greater availability of healthy foods, lower prices of healthy options, higher food quality, and a higher percentage of food that is labeled than CS. Moreover, we postulate that food stores from the food deserts and food swamps would have lower availability of healthy foods, a higher price of healthy options, lower food quality, and smaller portions of labeled foods than those from food oases.

## 2. Methods

### 2.1. Design of Food Auditing Instrument

High-quality food auditing instruments are critically essential to evaluate the consumer nutrition environment [1]. A common way to measure the consumer nutrition environment in the early studies was the standard food basket. A series of standard food baskets were developed [15,30,32,33,34,35,36]. However, these food basket instruments focus on specific food items (e.g., healthy foods) in a store. They do not allow for the comparison of food prices between healthy and regular options. Moreover, many studies did not report the reliability of their instruments [15,33,34,36].

The Nutrition Environment Measures Survey (NEMS) was developed as an observational tool to assess consumer nutrition environments [37]. It consists of a serial of different instruments, including NEMS-S (for stores) and NEMS-R (for restaurants), NEMS-CS (for corner stores), NEMS-V (for vending machines), and NEMS-P (for perceived nutrition environment). However, NEMS does not include some culturally appreciated foods that are local but very important to residents. Take Texas as an example; 35% of the population is Hispanic/Latino. The ethnic group has their own cultural identities and food preferences. Nutrition, Physical Activity and Obesity Prevention (NPAOP) developed a culturally sensitive tool and named it “Texas Nutrition Environment Assessment in Stores (TxNEA-S)” [20]. It is an adaptation of NEMS including additional foods that are culturally important to Hispanics and other minorities in Texas. Although TxNEA-S is useful and meaningful in Texas, it needs to be tailored for specific projects. First, TxNEA-S does not include sugar-sweetened beverages, which contain high calorie levels, potentially making people more obese. Second, it does not contain meat such as ground beef and chicken, which are critical protein sources for Americans. Third, TxNEA-S is excessively lengthy (i.e., 24 pages) and requires a lot of time to complete. It would take about two and a half hours to finish auditing one grocery store. For this study, it is necessary to modify TxNEA-S to focus on the items that are known as being essential for obesity-related food environment assessment.

The M-TxNEA-S food auditing tool was designed by customizing the TxNEA-S instrument. The primary food categories of the M-TxNEA-S include fresh fruit and vegetables, milk and cheese, grains, meat and meat alternatives, beverages, and snacks (i.e., chips and pretzels); canned and frozen fruits and vegetables are added because low-income people tend to purchase non-fresh options. The instrument consists of 10 pages and 93 food items. Each food item in the tool would be examined by availability, price, and labeling. Food quality is surveyed only for fresh fruits and vegetables. The M-TxNEA-S instrument is available upon request.

### 2.2. Selection of Surveying Neighborhoods in Austin, Texas

Austin, the capital of Texas, is in central Texas. It is one of the fastest-growing cities in the US [38]. Austin is facing food insecurity issues, especially in east Austin [38]. Nearly 25% of its residents live in urban food deserts. The past two decades have witnessed rapid population growth. This fast-growing population trend makes properties and housing values increase rapidly [38]. The increases in living costs force people to spend more money on rent rather than buying nutritious food; this phenomenon is more serious in east Austin [38]. According to the 2011 Sustainable Food Center (SFC) report, the values of a single-family home in the 78721 zip code had increased by 80% from 2000 to 2005 [39]. Moreover, east Austin is the most impoverished area in Travis County, and 40% of residents live below the federal poverty level [39]. Households in East Austin are not able to acquire enough food. Food stores in East Austin are generally limited not only in counts but also in size. Large and healthy food outlets are concentrated in wealthy neighborhoods on the west of Austin [39]; not to mention that there is a lack of reliable public transportation systems in east Austin, making low-income residents challenging to commute for grocery shopping.

**Figure 1:**
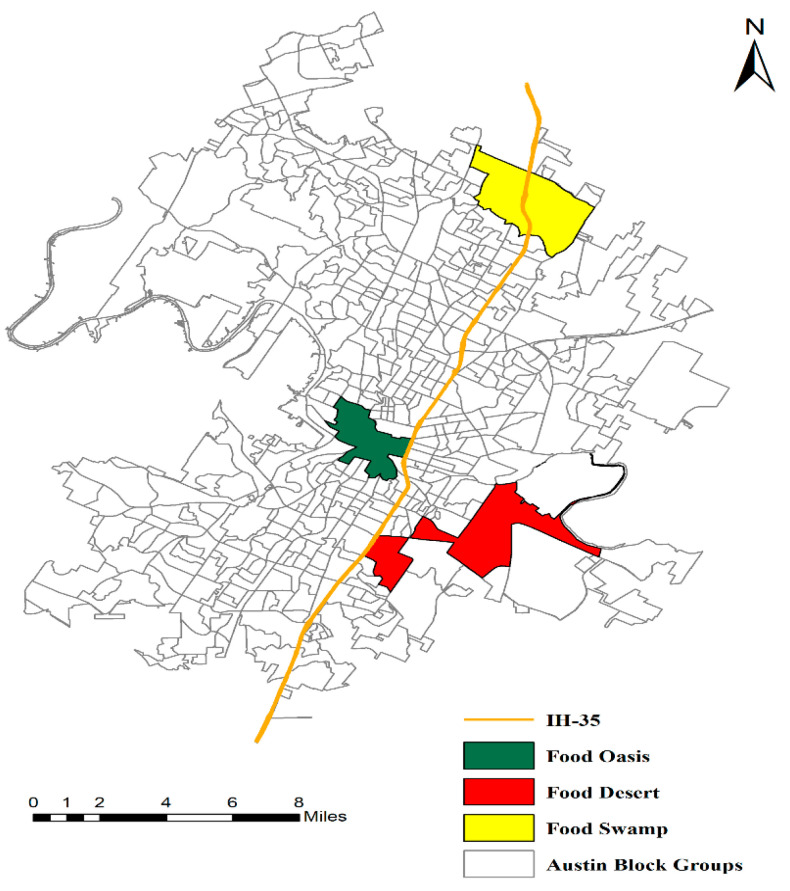
The neighborhoods of food deserts, food swamps, and food oases in Austin, Texas that are included for this study.

We, therefore, selected food deserts (FD) and food swamps (FS) in the impoverished areas of Austin to conduct the food survey (see Figure 1). The food deserts are on the eastern side of Interstate-35 (I-35); the food swamps are in the northeast of Austin. We also included food oases (FO) in the analysis for a comparison purpose. The identified food oasis neighborhood is on the western side of the I-35 near downtown Austin (Figure 1). Note that the selection of the three neighborhoods is not random; instead, it is based on a quantitative computation with considering the SES and spatial access to food outlets. We used a novel method SAR-Gi* to integrate geographic access to food outlets and sociodemographic status (SES) to identify the FD, FS, and FO [40]. FD were identified as neighborhoods with low SES and low geographical access to healthy food outlets; FS referred to a neighborhood with low SES and high access to unhealthy food outlets; FO were neighborhoods with high SES and high access to healthy food outlets [40]. Table 1 shows the basic demographic, economics, and social-cultural statistics of the three neighborhoods. The food oases had the highest population density, while the food deserts had the lowest. On average, the SES (reflected as economic and socio-cultural characteristics in Table 1) was low in the food deserts, medium in the food swamps, but high in the food oases.

### 2.3. Food Store Identification and Store Survey

We employed two methods to identify retail food stores in the three neighborhoods. Using the online directory database, Reference USA, we found a total of 42 food stores. Then, we employed Google Maps to search keywords including grocery store, supermarket, supercenter, convenience store, mini-mart, food store, retail food store, food mart, corner store, mom and pop store, and bodega. An additional eight food stores were identified. We then traveled to the three neighborhoods to verify the locations of the stores. The store identification between Reference USA and Google Maps and field trips almost corresponds to each other in both food desert and food swamp neighborhoods (Table 2). However, there are noticeable discrepancies for GS in the food oasis neighborhood. Note that we differentiated GS and CS based on North American Industry Classification System (NAICS) codes that were available in the Reference USA dataset. The field trips also enabled us to correct some of the store classification information obtained from Reference USA and Google Map. A total number of 46 food stores were selected to be surveyed within the three neighborhoods (Table 2). Among these 46 stores, 13 stores were in the food deserts, 17 were in the food swamps, and 16 were in the food oases, respectively. Note that the number of stores (e.g., 13) surveyed in the food deserts does not meet the standard that the sample size should be at least 15 [37]. Nevertheless, it did make sense, since food deserts were inherently short of food stores.

The survey was conducted during the week of 17–24 August 2019. The two raters entered a store simultaneously but conducted the survey independently by checking food items in different orders. If the inter-rater reliability for the raters in a store is below the threshold (i.e., 80%), one of the raters conducted a second survey until the reliability threshold is met. The two raters received professional training from the researchers about the M-TxNES-S; they then led a pilot assignment in a grocery store in San Marcos, Texas. Once their inter-rater reliability achieved 80% and above, the training was complete.

### 2.4. Data Analyses

All data analyses were conducted in SPSS 25.0 (IBM Corp., Armonk, NY, USA). For the two independent food surveys conducted in each of the food stores, the percentages of agreement for food availability, price, quality, and labeling were calculated, respectively, following Glanz et al. [37]. An item-by-item agreement between the two raters was examined and the percentage agreement—the frequency of correctly matched responses divided by the total number of observations—was calculated. Then, the availability of healthy foods score was calculated using the percentage values (i.e., the number of healthy food items was divided by 93 food items and then multiplied 100). A two-way ANOVA was utilized to examine whether the availability of healthy foods was different the two types of stores (ST, i.e., grocery stores and supermarkets and convenience stores) and across the three-neighborhood environment (NE, i.e., food deserts, food swamps, and food oases).

A paired *t*-test was conducted to compare the price between regular items and low-fat (or fat-free) alternatives. Some food items such as fresh fruits are sold by the pound in GS, but they are sold by the piece in CS. To make the prices comparable, we converted the price per piece to per pound. Price comparisons were calculated as a percentage, based on the average cost for a healthy item compared to its low-fat counterpart. There were 19 selected pairs of healthy and low-fat items, including pear (or peach or mixed fruits) in heavy vs. light syrup, whole milk vs. fat-free milk, regular vs. fat-free yogurt, regular vs. non-fat cheese, flour vs. corn tortilla, white vs. whole wheat bread (or pasta or cheerio), ground vs. lean beef, regular vs. skinless chicken breast, regular vs. diet coke, 100% juice vs. juice drink, and regular vs. low-fat chip (or pretzel).

For food quality analysis, visual check of the food items in a store was conducted. When more than 50% of a specific fruits and vegetables (F&V) (i.e., bananas) in a food store was found to be of acceptable, we circled an “A” on the food survey instrument; otherwise, we circled a “U” for unacceptable. Then, we calculated the percentage of F&V in acceptable quality for a store. The histogram of food quality data revealed that it was severely left-skewed. We applied nonparametric one-way ANOVA (Mann–Whitney U test, and Kruskal–Wallis H test) examine whether food quality shows significant difference across two store types and three different neighborhood environments.

Food items with nutrition fact labels in stores were marked as labelled on the food instrument; otherwise, “no labelled” were marked. We calculated the percentage of food with appropriate labels for each store, which was not normally distributed. Therefore, a non-parametric Friedman Two-way ANOVA analysis was adopted to compare the percentage of food labeling by neighborhoods and store types. We further performed a Two-way ANOVA after removing the data outlier (only one store) to (re)examine the effect of neighborhoods and store types on food labelling.

## 3. Results

Among the 46 stores, five stores (one Hispanic grocery store and four convenience stores) refused to participate in the survey, resulting in 13 GS, and 28 CS for analysis. The cross-tabulation of all GS and CS across the three neighborhoods revealed that the food swamp neighborhood had 150% as many CS as the food oasis neighborhood, and the food desert neighborhood had barely 30% as many GS as the food oasis neighborhood. About 62% (11 out of 13) of the GS had three or more cash registers; small grocery stores (with 1 or 2 cash registers) were more common in food desert neighborhoods, whereas 95% of CS had no more than three cash registers. Approximately 70% of food stores accepted food stamps, but only a few stores (i.e., 30%) accepted the Women, Infants, and Children (WIC) nutrition program. The mean time to complete the survey was 35.08 (SD 29.03) minutes for grocery stores and supermarkets and 6.84 (SD 2.45) minutes for convenience stores.

The inter-rater reliability assessment of healthy food availability, price, quality, and labeling is reported in Table A1 in the Appendix A. The agreement rates on the availability measure were consistently high, ranging from 91.70% to 100%. Food price had a moderate agreement rate, with a mean of 86.08% and a standard deviation of 5.78%. Food quality and food labeling both had a high agreement rate.

### 3.1. Healthy Food Availability

The mean number of healthy food items across the 41 food stores was 17.15, with a standard deviation of 21.94. The mean percentage of healthy food availability was 6.45 (SD = 18.82%). A two-way ANOVA analysis was conducted to examine the effect of store type (ST) and neighborhoods (NE) on healthy food availability. There was an interaction between the effects of ST and NE on the availability of healthy foods, suggesting that the difference of healthy foods availability in the different nutrition neighborhoods (NE) was dependent on store types (ST). There were also significant main effects of ST and NE on the healthy food availability. Reporting the main effects could be misleading when there was a statistically significant interaction (https://statistics.laerd.com/spss-tutorials/two-way-anova-using-spss-statistics.php, accessed on 8 October 2019). Therefore, we conducted a separate simple main effect analysis on the effects of ST and NE.

Analyses of the simple main effect for store type (ST) for each neighborhood environment (NE) were performed with Bonferroni adjustment and *p* < 0.025 level (this analysis involves the testing of multiple simple main effects. A common method is to apply a Bonferroni adjustment to the declared statistical significance (i.e., *p* = 0.05). We need to divide the current level (*p* = 0.05) by the number of simple main effects (in this case, 2). We declare a simple main effect as statistically significant if *p* < 0.025). All pairwise comparisons were run for each simple main effect. As shown in Table 3, in the food deserts, there was no significant difference in healthy food availability across the two types of stores. In the food swamps and food oases, a statistically significant difference was observed in the mean percentage of healthy food availability between GS and CS. Table 4 reports on and compares between the mean percentage of healthy food availability for GS and CS in Fd, FS, and FO, respectively. It can be seen that the GS in both FS and FO neighborhood carry significantly much higher percentage of healthy food items than the CS. However, in the food deserts, the difference between GS and CS in their percentage of healthy food carrying disappeared.

**Figure 2:**
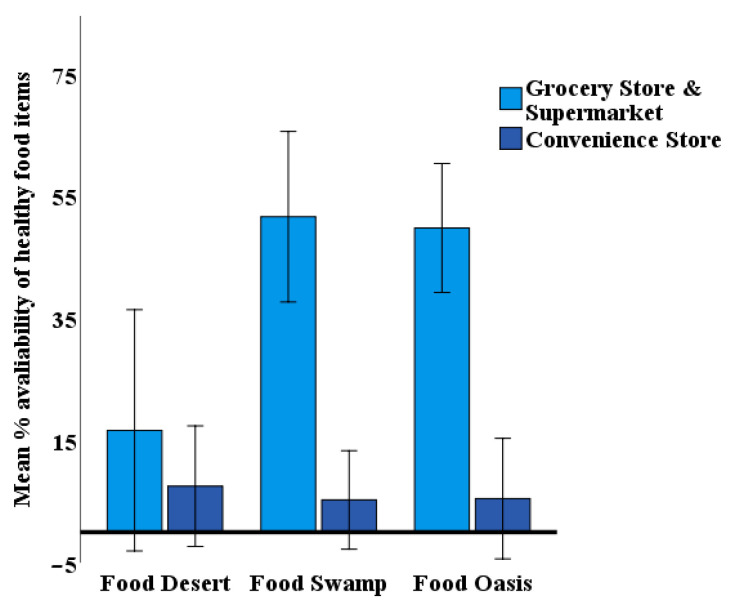
The difference in the mean percentage of healthy food availability by ST and NE.

Figure 2 illustrated the simple main effect of store type in different neighborhoods. Error bars showed the upper and lower 97.5% confidence intervals that extend above and below the mean column. It was evident that GS and CS had substantial differences in food swamp and food oasis neighborhoods (i.e., non-overlapping CI) in healthy food availability, while the difference was trivial in food desert neighborhoods. The graph confirms the appropriateness of analyzing the simple main effect of store type (ST) in this research.

**Figure 3:**
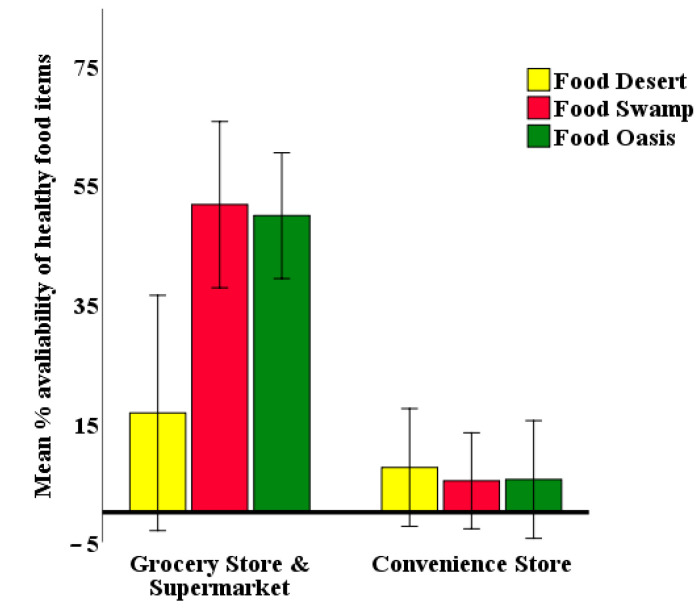
The difference in the mean percentage of healthy food availability by NE and ST.

An analysis of the simple main effect for the different NE at each store type was also performed with a Bonferroni adjustment (*p* < 0.025) level. A statistically significant difference was found in mean percentage of healthy food availability in the GS across food deserts, food swamps, and food oases (F (2, 35) = 5.13, *p* = 0.01, partial η^2^ = 0.23), but there was no significant difference for convenience stores across the three neighborhoods (F (2, 35) = 0.07, *p* = 0.932, partial η^2^ = 0.00) (see Table A2 in the Appendix A). Mean percentages of healthy food availability in the GS stores in the food deserts, food swamps, and food oases were 16.67, 51.61, and 49.77 (Table A3 in the Appendix A), respectively. GS in the food deserts had a significantly lower level of healthy food availability than those in food swamps and in the food deserts. Figure 3 shows the simple main effect of NE in different types of food stores. GS in the food swamp and food oasis neighborhoods had substantial differences regarding healthy food availability since there were non-overlapping CI, which also justified the appropriateness of examining the simple main effect of NE.

### 3.2. Food Price

Table 5 showed that the prices for most of the low-fat (i.e., lower-fat, lower-calorie, and whole-grain) items were not significantly different from those of the corresponding regular option items (14 out of 19 pairs, *p* > 0.05). The remaining five pairs had significant differences between the regular and alternative options (*p* < 0.05). The mean prices for mixed fruit in light syrup was 109.60% of the heavy syrup (*p* = 0.04); whole wheat bread was 106.30% of its white counterpart (*p* = 0.03). The most significantly substantial differences were found in the higher cost of skinless chicken breast, lean beef, and low-fat chips.

### 3.3. Food Quality

**Figure 4:**
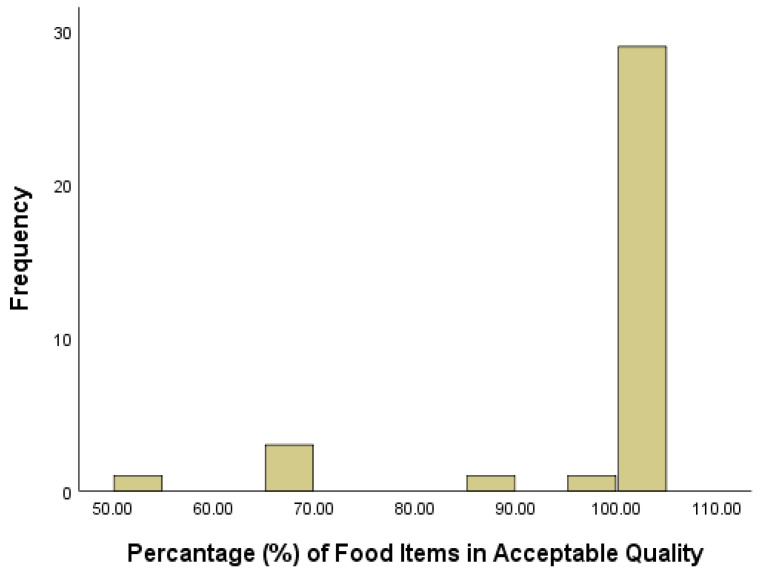
Histogram of percentage of food items in acceptable quality.

The percentages of F&V in acceptable quality in the 35 food stores ranged from 50% to 100%. However, the values of the 1st quartile, median, and third quantile were all 100%. This indicates that most of the food stores had a high percentage of acceptable F&V. The histogram was severely left-skewed, and 30 out of 35 stores had 100% F&V in acceptable quality (Figure 4). Since the dependent variable was not normally distributed (Shapiro–Wilk test = 0.44, df = 38, *p* = 0.000), and the various transformation techniques (square root, natural logarithm, logarithm, and reciprocal) were not able to produce a normal distribution, we performed a nonparametric one-way ANOVA on the percentage of F&V in acceptable quality by ST and NE, respectively. A Mann–Whitney U test was conducted on the quality percentage by ST, and a Kruskal–Wallis H test was performed by NE. There was no significant difference between ST (e.g., *p* = 0.80) nor NE (e.g., *p* = 0.27) in quality percentage.

### 3.4. Food Labeling

Forty out of the 41 food stores had more than 75% of the food items properly labeled the nutrition fact information. The store with only 62.5% of its food items labeled seems to be an outlier. Since the data are not normally distributed, we used a Friedman two-way ANOVA to perform the analysis, and it revealed no significant differences in food labeling by store type or neighborhood (chi-square = 8.39; *p* = 0.08). In addition, the histogram was normally distributed (Shapiro–Wilk test = 0.95, df = 40, *p* = 0.11) after eliminating this outlier. We performed a two-way ANOVA analysis on the percentage of food labeling for 40 stores by ST and NE. There was neither a significant interaction of ST and NE (F (2, 34) = 0.65, *p* = 0.53) nor a main effect on food labeling (F (1, 34) = 0.90, *p* = 0.35; F (2, 34) = 0.46, *p* = 0.46). The same results are held by a parametric two-way ANOVA when the outlier is excluded.

## 4. Discussion

We developed the M-TxNEA-S by customizing the original TxNEA-S to measure the nutrition environment in Austin, Texas. The M-TxNEA-S instrument was utilized in this study due to several reasons. First, the M-TxNEA-S instrument contains a series of food items that are culturally important to the ethnic groups, such as Hispanics/Latinos in Texas, and hence reflects residents’ dietary behaviors and food preferences. Second, we reduced the redundancy of the original TxNEA-S by removing several food items that are relatively less important to Texans’ diet. Meanwhile, we added meats (i.e., beef and chicken breast) and beverages that are consumed by many residents in Texas regularly. Third, in addition to the assessment of food availability, price, and quality that are commonly evaluated by the existing food instruments, we added food labelings in the instrument. Proper food labelings are believed to impose a positive influence on people’s food purchasing behaviors.

The M-TxNEA-S has shown high inter-rater reliability, suggesting that the protocols and instructions are valid and are sound enough to prepare data raters to collect reliable data. It also complies with Glanz et al.’s [37] recommendation that the modifications of NEMS-S and its variants must evaluate the reliability. Compared to the studies that failed to test reliability [15,33,34,36], the use of inter-rater reliability in this study has a distinct advantage. Our survey tool (M-TxNEA-S) was compared with the TxNEA-S [20]; in general, the reliability scores of our tool were higher. In addition, the inter-rater reliability for food availability, price, quality, and labels were all reported, while TxNEA-S only reported the inter-rater reliability of food availability.

Previous studies often focused on whether healthy foods are less available in low-income or minority neighborhoods [1,21,41,42]; no studies to date have paid attention to the differences in food availability across different types of neighborhoods. To the extent of our knowledge, this is the first to examine and compare the consumer nutrition environment across the food deserts, food swamps, and food oases. The result shows that the nutrition environment, as defined by healthy food availability, was highly related to the different types of food stores in such a neighborhood. Specifically, the grocery stores in food swamp and food oasis neighborhoods had significantly higher healthy food availability than the convenience stores, but this was not the case for food desert neighborhoods; the grocery stores in food deserts are small and have a limited range of healthy foods. Although these stores are named grocery stores, they are essentially like convenience stores. Therefore, the difference in the nutrition environment by store types does not apply to food desert neighborhoods.

In this study, food deserts were in low SES areas, food swamps were in medium SES areas, and food oases were in high SES areas. The findings thus support the previous results that healthy foods are less available in grocery stores in low-income neighborhoods [1,2,21]. However, it does not align with Gloria and Steinhardt’s [20] finding that income has a positive relationship with healthy food availability. In our study, the food swamp neighborhood had a low to medium SES but had the highest mean healthy foods availablity in grocery stores. We found that the food swamps have excessive access to unhealthy foods as well as high access to healthy foods.

Regarding the price comparison between the regular and low fat (or fat-free) food options, our findings are consistent with Glanz et al. [2] that the price for most healthy choices is not significantly different than their regular alternatives. Only a few items, such as low-fat meats (i.e., beef and chicken breast), whole wheat bread, and low-fat chips, are priced higher than the regular counterparts, corresponding to Glanz and colleagues’ findings. However, our study did not find any significant price difference between 100% juice and juice drink. Healthy food was found to be cheaper than regular counterparts in food desert neighborhoods and convenience stores. However, the price difference was minimal across store types and communities.

The quality of F&V across different stores was consistently high; this is consistent with the previous research claiming that products in food stores are generally of high quality [1]. Furthermore, we found no significant difference in the quality of F&V across different neighborhoods, which is consistent with Coulter [1]. Nevertheless, it does not support Cummins et al.’s results that medium-sized stores in a high-SES neighborhood had the highest quality fresh products [43]. Nor does our finding align with Glanz et al.’s conclusion that grocery stores in high-income neighborhoods had high-quality foods [2]. Our results do not support the findings that F&V quality is different by store type and neighborhood marginalization [41,44].

This research focused on in-store characteristics aiming at improving the understanding of the consumer nutrition environment, and the findings can shed light on designing intervention programs in food-insecure neighborhoods. Specifically, the availability of healthy food in food deserts was not significantly different in GS and CS. The GS in food deserts are small groceries carrying a limited range of fruits and vegetables, beverages, and milk; they often are similar to CS. If the establishment of large food stores in food desert neighborhoods is not economically rewarding, setting up more small groceries with better healthy options might alleviate food security issues. In addition, it is informative to learn that GS in food desert neighborhoods should increase their healthy food availability, keep their food price low, and improve food quality and labeling. GS in food swamps had the highest healthy food availability among the three neighborhoods, suggesting that the residents in this neighborhood are likely to procure enough healthy foods and eat healthily. The food swamp designation of these neighborhoods might be more related to the overwhelming presence of unhealthy food environments, especially fast-food restaurants. Stakeholders should consider intervention strategies to control the development of fast-food restaurants.

The findings from this study help to reveal the different roles that healthy food availability, affordability, quality, and labeling may play for the consumer nutrition environment, which further impacts individuals’ purchasing behavior and diet choice. The healthy food availability did vary significantly across the neighborhoods and the store types, but food price, food quality, and food labeling did not. One possible explanation is that the aggregation of data may smooth out the variation. Nevertheless, the interpretation of this finding is challenging because the deprivation amplification theory does not seem to uphold healthy food availability in the food swamp neighborhood. That being said, residents from deprived neighborhoods are not necessarily always suffer from a poor food environment [3]; some deprived areas may have good access to healthy foods. This articulation is still controversial in food studies because of the various ways of characterizing the food environment. The argument also motivates researchers to put more effort into exploring to what extent the social deprivation influences residents’ access to different food options.

This study is not without limitations. First, the sample size for food stores is limited. The sample size of grocery stores (13 stores) and convenience stores (28 stores) do impact the interpretation power of the findings. Second, due to the financial budget, the data are from a one-time survey with no revisit to the same stores. Some studies conducted the survey twice, and the second survey is held two or three weeks after the first one [2,20]. The two-survey approach allows test-retest reliability examination so that the stability of the stores’ food environment can be assessed. We will conduct two surveys when external funding is available in the future. Third, the survey was done only in the summer season. It does not capture the price and quality variations across different seasons.

## 5. Conclusions

We examined the consumer nutrition environment in the food deserts, food swamps, and food oases in Austin, Texas by creating a new food survey instrument to better represent residents’ dietary cultural preferences in the southern United States. The consumer nutrition environment usually contains food availability, price, and quality. Our research extended it and included food labeling, which has been underexamined by other studies. We found that grocery stores had a significantly higher availability of healthy foods than convenience stores across the neighborhoods, except for the food deserts. For grocery stores, the mean percentage of healthy food availability in food swamps and food oases was significantly higher than the food deserts; food swamps and food deserts had the highest and lowest percentages of healthy food availability, respectively. For convenience stores, food deserts had the highest healthy food availability, but the difference was insignificant between the three neighborhoods. We also found that the price for most of the healthy items was not significantly different from their regular counterparts. For food quality and labeling, there was not any significant difference between store types and neighborhoods. In summary, store type and neighborhood nutrition environment are both significantly related to healthy food availability, but not related to food price, quality, and labeling in our study. This finding suggests that effective intervention aiming at providing equal access to healthy foods in impoverished neighborhoods should emphasize an increase in healthy food availability, not price or quality or labeling of healthy food items. In addition, our analyses revealed that the grocery stores and supermarkets in food deserts do not carry more healthy foods than the convenience stores. As major suppliers for healthy foods in the neighborhoods of food oases and food swamps, these grocery stores and supermarkets have the potential to do the same in impoverished neighborhood. Future studies should examine what may be hindering these stores from doing so. Our study is helpful for practitioners and policy makers to identify policies and incentive programs that can increase healthy food supply in the grocery stores and supermarkets in food desert neighborhoods.

## Figures and Tables

**Table 1:** General characteristics of the three neighborhoods in Austin, Texas.

-Characteristics	Food Deserts	Food Swamps	Food Oases
The number of Block Groups	13	12	13
Total population	18,173	17,439	16,945
Size (square miles)	8.85	6.48	3.82
Density (# people/sq. mi)	2053.57	2691.68	4437.83
Economic Characteristics	Mean ± SD	Mean ± SD	Mean ± SD
Household income ($1000)	33.53 ± 8.31	49.40 ± 6.78	75.69 ± 16.12
Below poverty line (%)	31.60 ± 9.84	8.28 ± 3.52	6.41 ± 4.71
Lack of kitchen facility (%)	2.14 ± 3.41	1.17 ± 2.49	2.52 ± 3.85
Sociocultural Characteristics	Mean ± SD	Mean ± SD	Mean ± SD
Without Higher Education (%)	89.10 ± 9.43	61.50 ± 12.38	18.74 ± 4.65
Hispanic people (%)	79.32 ± 13.97	35.82 ± 15.09	9.20 ± 3.79
Language isolation (%)	0.15 ± 0.08	0.06 ± 0.04	0.02 ± 0.03

Comments: GS: grocery stores and supermarkets; CS: convenience stores; SD: Standard Deviation.

**Table 2:** The number of food stores identified in the three selected neighborhoods.

Neighborhood	Store Identification Methods	GS	CS	Total
FD	R-USA and GM	3	9	12
	FT	3	10	13
FS	R-USA and GM	5	12	17
	FT	4	13	17
FO	R-USA and GM	12	10	22
	FT	7	9	16

Comments: FD: Food Deserts; FS: Food Swamps; FO: Food Oases; R-USA and GM: Reference USA and Google Maps; FT: Field Trip.

**Table 3:** Simple main effects of GS and CS within each of the three types of NE.

NE	Test-	Sum of Squares	DF	Mean Square	F	*p*	Partial η^2^
FD	Contrast	133.66	1	133.66	0.71	0.41	0.02
	Error	6613.16	35	188.95			
FS	Contrast	6438.34	1	6438.34	34.08	0.00 **	0.49
	Error	6613.16	35	188.95			
FO	Contrast	7313.02	1	7313.01	38.70	0.00 **	0.53
	Error	6613.16	35	188.95			

Comments: FD: Food Deserts; FS: Food Swamps; FO: Food Oases; DF: Degree of Freedom.*: significant at α = 0.05 level; **:significant at α = 0.01 level.

**Table 4:** Multiple comparisons on the percentages of GS and CS that carry heathy food in each of the three types of NE.

	GS (I): Mean ± SE	CS (J): Mean ± SE	Difference (I)–(J): Mean ± SE	*p*	97.5% CI for Difference ^a^
FD	16.67 ± 9.72	7.53 ± 4.86	9.14 ± 10.87	0.41	(−16.31, 34.59)
FS	51.61 ± 6.87	5.29 ± 3.97	46.33 ± 7.94	0.00 **	(27.74, 64.91)
FO	49.77 ± 5.20	5.52 ± 4.86	44.26 ± 7.11	0.00 **	(27.60, 60.92)

**:significant at α = 0.01 level. Comments: ^a^: Bonferroni adjustment for multiple comparisons.

**Table 5:** Paired *t*-test for healthy vs. regular items.

-Food Items in Pairs	Healthy Item (I)		Low-Fat Item (J)		(I)/(J) × 100	Difference of (I) − (J)		*p*
-Statisistics	Mean	SD	Mean	SD		Mean	SD	
Pear in syrup	0.10	0.03	0.09	0.04	109.00	0.01	0.01	0.24
Peach in syrup	0.09	0.04	0.08	0.03	123.10	0.01	0.02	0.42
Mixed Fruit in syrup	0.12	0.04	0.11	0.04	109.60	0.01	0.01	0.04 *
Milk (128 oz)	3.91	0.87	4.08	0.92	95.72	−0.18	0.69	0.19
Milk (64 oz)	2.92	0.94	3.03	0.86	96.31	−0.11	0.39	0.17
Milk (32 oz)	1.92	0.41	1.94	0.42	99.17	−0.02	0.08	0.32
Lactose Free (64 oz)	4.57	1.50	3.77	0.78	121.30	0.80	0.99	0.08
Yogurt	0.20	0.25	0.20	0.25	80.92	0.00	0.03	0.88
Cheese	0.15	0.13	0.12	0.06	126.10	0.03	0.07	0.37
Tortilla	0.15	0.06	0.17	0.08	88.60	−0.02	0.06	0.20
Bread	0.15	0.06	0.14	0.06	106.30	0.01	0.01	0.03 *
Pasta	0.29	0.41	0.23	0.31	126.90	0.06	0.11	0.14
Cheerio	0.24	0.12	0.26	0.09	90.05	−0.03	0.04	0.37
Beef	0.32	0.07	0.24	0.06	135.50	0.08	0.03	0.00 **
Chicken Breast	0.21	0.08	0.13	0.05	153.35	0.07	0.06	0.04 *
Coke	0.08	0.04	0.08	0.04	100.00	NA	NA	NA
Juice	0.12	0.07	0.13	0.07	96.18	−0.01	0.03	0.41
Chip	0.53	0.19	0.43	0.15	124.90	0.11	0.16	0.00 **
Pretzel	0.42	0.22	0.52	0.23	80.80	−0.10	0.16	0.06

Comments: NA: not applicable for the price comparsion between regular and diet coke due to the same price on average. *: significant at α = 0.05 level; **:significant at α = 0.01 level.

## Data Availability

The survey data are available upon request from the corresponding author.

## Appendix A

**Table A1:** Inter-rater reliability for the M-TxNEA-S food instrument.

Store ID	ST	NNE	Inter-Rater Reliability			
			Availability	Price	Quality	Label
001-001-01	CS	FD	0.95	0.86	1.00	0.91
001-002-01	GS	FD	0.96	0.85	1.00	0.96
001-002-02	GS	FD	0.92	0.92	0.92	0.96
001-004-02	CS	FD	1.00	1.00	1.00	1.00
001-004-03	CS	FD	1.00	0.90	1.00	0.95
001-004-04	CS	FD	0.93	0.87	1.00	0.93
001-004-05	CS	FD	0.93	0.87	1.00	0.87
001-004-06	CS	FD	1.00	0.81	1.00	0.92
001-004-07	CS	FD	1.00	0.93	1.00	0.93
001-004-09	CS	FD	0.95	0.90	1.00	0.95
002-001-01	GS	FS	0.98	0.87	1.00	0.93
002-001-02	GS	FS	0.95	0.85	1.00	0.85
002-001-03	GS	FS	1.00	0.87	0.95	0.98
002-001-04	CS	FS	0.92	0.75	0.83	0.92
002-003-01	GS	FS	0.99	0.86	1.00	0.96
002-004-01	CS	FS	1.00	0.89	1.00	0.94
002-004-02	CS	FS	1.00	0.89	NA	1.00
002-004-03	CS	FS	1.00	0.85	1.00	0.92
002-004-05	CS	FS	1.00	0.85	1.00	0.92
002-004-06	CS	FS	1.00	0.86	1.00	0.86
002-004-07	CS	FS	1.00	0.89	NA	1.00
002-004-08	CS	FS	0.92	0.85	NA	0.92
002-004-09	CS	FS	1.00	0.88	NA	1.00
002-004-10	CS	FS	1.00	0.82	1.00	0.82
002-004-11	CS	FS	0.93	0.80	NA	0.93
002-004-12	CS	FS	0.92	0.85	NA	0.92
003-001-02	GS	FO	0.99	0.80	1.00	0.87
003-001-04	CS	FO	1.00	0.87	NA	0.93
003-001-05	GS	FO	1.00	0.91	1.00	0.95
003-001-06	GS	FO	1.00	0.84	1.00	0.97
003-001-07	GS	FO	0.97	0.79	1.00	0.94
003-001-08	GS	FO	0.99	0.79	1.00	0.96
003-001-09	GS	FO	0.99	0.80	1.00	0.98
003-001-11	GS	FO	1.00	0.80	1.00	0.99
003-004-02	CS	FO	0.93	0.87	1.00	0.80
003-004-03	CS	FO	1.00	0.88	NA	1.00
003-004-04	CS	FO	0.94	0.89	NA	0.83
003-004-05	CS	FO	1.00	0.75	NA	1.00
003-004-07	CS	FO	1.00	1.00	NA	1.00
003-004-08	CS	FO	1.00	1.00	NA	1.00
003-004-10	CS	FO	1.00	0.80	NA	1.00

Comment: NA: not applicable due to few (i.e., ≤ 3) fresh fruits and vegetables in that store.

**Table A2:** Simple main effects of FD, FS, and FO within each of the two types of stores.

ST	Test	Sum of Squares	DF	Mean Square	F	*p*	Partial η^2^
GS	Contrast	1938.95	2	969.47	5.13	0.01 *	0.23
	Error	6613.16	35	188.95			
CS	Contrast	26.67	2	13.33	0.07	0.93	0.00
	Error	6613.16	35	188.95			

Comment: *: significant at α = 0.05 level.

**Table A3:** Multiple comparisons on the percentages of FD, FS, and FO that carry heathy foods in each of the two tyeps of stores.

ST	(I) NNE: Mean ± SE	(J) NNE: Mean ± SE	Difference (I)–(J): Mean ± SE	*p*	97.5% CI for the Difference
GS	FD: 16.67 ± 9.72	FS: 51.61 ± 6.87	−34.95 ± 11.90	0.02 *	(−68.24, −1.65)
	FS: 51.61 ± 6.87	FO: 49.77 ± 5.20	1.85 ± 8.62	1.00	(−22.25, 25.94)
	FO: 49.77 ± 5.20	FD: 16.67 ± 9.72	33.10 ± 11.02	0.02 *	(2.28, 63.93)
CS	FD: 7.53 ± 4.86	FS: 5.29 ± 3.97	2.24 ± 6.27	1.00	(−15.30, 19.79)
	FS: 5.29 ± 3.97	FO: 5.51 ± 4.86	−0.22 ± 6.27	1.00	(−17.77, 13.32)
	FO:5.51 ± 4.86	FD: 7.53 ± 4.86	−2.01 ± 6.87	1.00	(−21.13, 17.20)

Comment: *: significant at α = 0.05 level.
